# P-1928. Virologic and Clinical Outcomes Among Participants Hospitalized in a Phase 3 Trial Comparing Molnupiravir with Placebo for Treating Mild-to-Moderate COVID-19

**DOI:** 10.1093/ofid/ofae631.2088

**Published:** 2025-01-29

**Authors:** Matthew G Johnson, David W Hilbert, Erin Jensen, Dorothy McCoy, Brian Maas, Ying Zhang, Michelle L Brown, Dana L Byrne, Carisa S De Anda

**Affiliations:** Merck & Co., Inc., Rahway, New Jersey; Merck Research Laboratories, Rahway, New Jersey; Merck & Co., Inc., Rahway, New Jersey; Merck & Co., Inc., Rahway, New Jersey; Merck & Co., Inc., Rahway, New Jersey; Merck & Co., Inc., Rahway, New Jersey; Merck & Co., Inc., Rahway, New Jersey; Merck & Co., Inc., Rahway, New Jersey; Merck & Co., Inc., Rahway, New Jersey

## Abstract

**Background:**

The correlation between virologic and clinical outcomes in COVID-19 remains unclear. The objective of this analysis was to assess SARS-CoV-2 viral load (VL) and laboratory biomarkers in participants who did and did not progress to hospitalization in the phase 3 component of the MOVe-OUT trial.

**Table 1.**
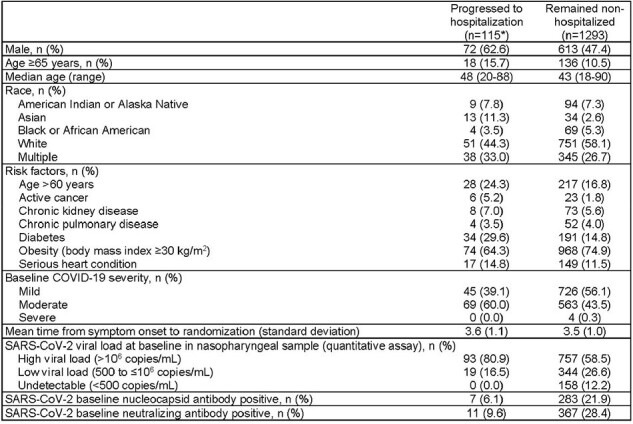
Baseline demographics and characteristics of participants who did and did not progress to hospitalization *One participant in the placebo group with an unknown hospitalization status was not included in this analysis.

**Methods:**

In MOVe-OUT, unvaccinated adults with mild-to-moderate COVID-19 at risk for disease progression were randomized 1:1 to molnupiravir (MOV) 800-mg or placebo (PBO) every 12 hours for 5 days. The primary efficacy endpoint was all-cause hospitalization or death through day 29. In this analysis, the following were evaluated in the modified intent-to-treat (MITT; randomized, received ≥1 dose of study drug, and not hospitalized prior to 1^st^ dose) population (1408 participants) at baseline and at postbaseline time points: VL (quantitative PCR), high-sensitivity C-reactive protein (CRP), and absolute lymphocyte count (ALC).

**Figure 1.**
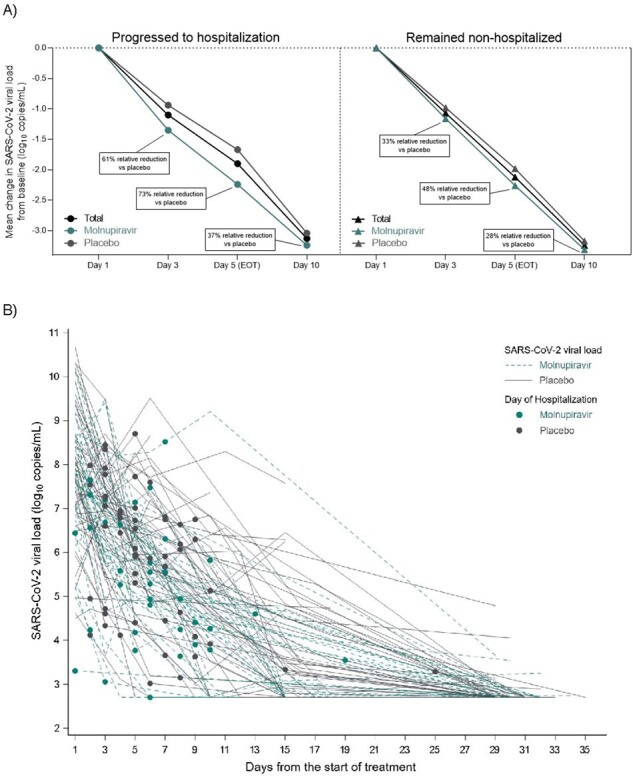
Mean change in SARS-CoV-2 viral load in hospitalized and non-hospitalized participants (A) and SARS-CoV-2 viral load over time in hospitalized participants (B) Hospitalization ± 48 hours excluding baseline. One participant in the placebo group with an unknown hospitalization status was not included in this analysis.

**Results:**

115 (8.2%) participants progressed to hospitalization (HP). HP were more likely vs those who remained non-hospitalized (NHP) to be male; ≥65 years; with active cancer, diabetes mellitus, moderate COVID-19, and negative nucleocapsid and neutralizing antibody status at baseline (Table 1). Mean time from symptom onset to hospitalization, and randomization to hospitalization, was 8.9 and 5.3 days. In HP, mean VL at baseline was 7.51 vs 6.83 log_10_ copies/mL among NHP. Among HP, VL declined in 37/38 (97.4%) MOV and 50/63 (79.4%) PBO-treated participants, respectively, between baseline and the day of hospitalization. Mean decline in VL from baseline to day 5 was 1.90 (SD 1.53) in HP vs 2.12 (SD 1.62) log_10_ copies/mL in NHP, with greater declines observed in MOV vs PBO-treated participants at all time points (Figure 1). Mean CRP was higher at baseline in HP vs NHP (49.93 vs 16.77 mg/L), and postbaseline values increased in HP and decreased in NHP. Alternatively, mean ALC was lower at baseline in HP vs NHP (1.07 vs 1.54 10^9^/L), and postbaseline values continued to decrease at day 3 and 5 in HP while values increased in NHP (Table 2).

**Table 2.**
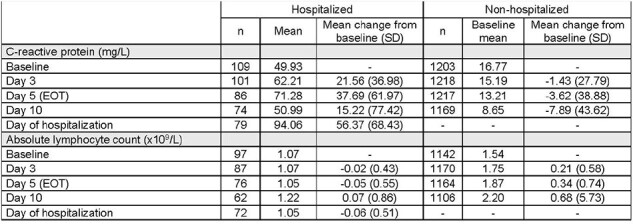
Mean change in C-reactive protein and absolute lymphocyte count in hospitalized and non-hospitalized participants Hospitalization ± 48 hours excluding baseline.

**Conclusion:**

HP had a higher VL at baseline, with a smaller decline over time than NHP. A higher VL at baseline in conjunction with an increasing CRP and decreasing ALC may help identify patients with mild-to-moderate COVID-19 at higher risk for hospitalization.

**Disclosures:**

Matthew G. Johnson, MD, Merck & Co., Inc.: I have an interest in a patent application for the company.|Merck & Co., Inc.: Employee of Merck & Co., Inc.|Merck & Co., Inc.: Stocks/Bonds (Public Company) David W. Hilbert, PhD, Merck & Co., Inc.: Employee of Merck & Co., Inc.|Merck & Co., Inc.: Stocks/Bonds (Public Company) Erin Jensen, MS, Merck & Co., Inc.: Employee of Merck & Co., Inc.|Merck & Co., Inc.: Stocks/Bonds (Public Company) Dorothy McCoy, PharmD, Merck & Co., Inc.: Employee of Merck & Co., Inc.|Merck & Co., Inc.: Stocks/Bonds (Public Company) Brian Maas, PharmD, Merck & Co., Inc.: Employee of Merck & Co., Inc.|Merck & Co., Inc.: Stocks/Bonds (Public Company) Ying Zhang, PhD, Merck & Co., Inc.: Employee of Merck & Co., Inc.|Merck & Co., Inc.: Stocks/Bonds (Public Company) Michelle L. Brown, BS, Merck & Co., Inc.: Employee of Merck & Co., Inc.|Merck & Co., Inc.: Stocks/Bonds (Public Company) Dana L. Byrne, MD, Merck & Co., Inc.: Employee of Merck & Co., Inc.|Merck & Co., Inc.: Stocks/Bonds (Public Company) Carisa S. De Anda, PharmD, Merck & Co., Inc.: MOV patent|Merck & Co., Inc.: Employee of Merck & Co., Inc.|Merck & Co., Inc.: Stocks/Bonds (Public Company)

